# Human meniscus allograft augmentation by allogeneic mesenchymal stromal/stem cell injections

**DOI:** 10.1002/jor.25074

**Published:** 2021-05-24

**Authors:** Caroline Struijk, Wouter Van Genechten, Peter Verdonk, Aaron J. Krych, Allan B. Dietz, Andre J. van Wijnen, Daniel B. F. Saris

**Affiliations:** ^1^ Orthopedics and Sports Medicine Mayo Clinic Rochester Minnesota USA; ^2^ Department of Orthopedic Surgery Antwerp University Antwerp Belgium; ^3^ ORTHOCA Antwerp Belgium; ^4^ Department of Laboratory Medicine and Pathology IMPACT; Mayo Clinic College of Medicine and Science Rochester Minnesota USA; ^5^ Orthopaedic Surgery University Medical Center Utrecht Utrecht The Netherlands; ^6^ Reconstructive Medicine University of Twente Enschede The Netherlands

**Keywords:** allograft, knee, meniscus, mesenchymal stromal cell, viability

## Abstract

Meniscus allograft transplantations (MATs) represent established surgical procedures with proven outcomes. Yet, storage as frozen specimens and limited cellular repopulation may impair graft viability. This proof‐of‐concept study tests the feasibility of injecting allogeneic mesenchymal stromal/stem cells (MSCs) in meniscus allograft tissue. We investigated the injectable cell quantity, survival rate, migration, and proliferation ability of MSCs up to 28 days of incubation. In this controlled laboratory study, seven fresh‐frozen human allografts were injected with human allogeneic MSCs. Cells were labeled and histological characteristics were microscopically imaged up to 28 days. Mock‐injected menisci were included as negative controls in each experiment. Toluidine blue staining demonstrated that a 100‐µl volume can be injected while retracting and rotating the inserted needle. Immediately after injection, labeled MSCs were distributed throughout the injection channel and eventually migrated into the surrounding tissues. Histological assessment revealed that MSCs cluster in disc‐like shapes, parallel to the intrinsic lamination of the meniscus and around the vascular network. Quantification showed that more than 60% of cells were present in horizontally injected grafts and more than 30% were observed in vertically injected samples. On Day 14, cells adopted a spindle‐shaped morphology and exhibited proliferative and migratory behaviors. On Day 28, live/dead ratio assessment revealed an approximately 80% cell survival. The study demonstrated the feasibility of injecting doses of MSCs (>0.1 million) in meniscus allograft tissue with active cell proliferation, migration, and robust cell survival.

## 1 INTRODUCTION

The knee menisci play a vital role in joint function and primarily contribute to load transmission, shock absorption, stability, and nutrient diffusion in the knee.^1^, ^2^ The C‐shaped meniscus is composed of water (72%) and a densely packed collagen network of mainly type 1 (90%) and type 2 collagen fibers.^1^ Compared with hyaline cartilage, proteoglycan content is relatively low but nevertheless important in determining viscoelastic properties of the meniscus.^1^, ^2^

Meniscal tears are the most common knee injury and frequently cause functional impairment and altered joint homeostasis, which ultimately results in a higher likelihood for early osteoarthritic changes.^3^ Due to the poor vascularization in the white zone,^4^ the natural healing capacity of avascular meniscal tears is relatively limited, thus justifying surgical intervention.^5^ Arthroscopic partial meniscectomy remains the most commonly performed knee surgery when a meniscal repair seems unfeasible.^6^, ^7^ Nevertheless, the resected meniscus area should be limited to the absolute minimum, because resection is directly proportional to increased peak contact forces and early degeneration of the articular cartilage.^8^, ^9^

After meniscectomy, a reasonable number of patients remain symptomatic or develop joint dysfunction and/or pain. Young patients without arthritis are suitable candidates for meniscus allograft transplantation (MAT). Best outcomes after MAT are reported in relatively younger patients (<50 years) with a stable and well‐aligned knee joint and minor articular cartilage degeneration.^10^ Overall graft survival rates are currently estimated as 73.5% at 10 years and 60.3% at 15 years, with a mean time to failure of approximately 7.8 years.^11^, ^12^ The ultimate goal is to restore knee biomechanics and improve functional outcome in patients, while providing sufficient chondroprotection to delay cartilage degeneration. There is no consensus on the chondroprotective value of MAT, but it is unlikely to be equivalent to a native intact meniscus.^13^, ^14^

Solicitation of expert opinions through a survey from the International Meniscus Reconstruction Experts Forum (IMREF) showed that the majority of surgeons (68%) prefer fresh‐frozen (−80°C) over cryopreserved meniscus allografts, mainly for logistical reasons and the relatively low associated cost.^15^ Current donor cell survival rates in cryopreserved grafts range from 4% to 54%,^11^ whereas meniscus cells do not survive fresh‐frozen storage at −80°C. Therefore, insertion of a “dead” acellular piece of donor tissue provides a plausible explanation for tissue degradation with concomitant graft shrinkage (65% minimal, 20% mild, and 16% moderate) in the first 3 months posttransplantation as well as graft tearing and extrusion.^16^, ^17^, ^18^, ^19^ These complications pose a serious concern, because preoperative graft sizing is already challenging and it could further deteriorate the biomechanical environment of the knee joint and reduce the chondroprotective effect.^16^, ^17^, ^18^, ^19^

Rapid cellular repopulation after implantation may mitigate the initial lack of viable cells in the graft, but this notion does not conform to empirical observation. Due to the densely packed collagen fiber network, cell migration into the graft is time‐consuming and largely incomplete. Rodeo et al.^20^ have presented histology findings demonstrating that only a thin layer of the grafts' contact surface contains cells from the synovial membrane (fibroblasts) and signs of remodeling at 6 months after transplantation.

Although the importance of initial meniscus allograft viability to outcome and durability remains unclear,^10^, ^13^ early remodeling and biological incorporation of meniscus grafts may facilitate favorable outcomes, patient rehabilitation and return to activities of daily living, work, and sports activities. The development of improved integration strategies using cell‐based procedures may repopulate and remodel meniscal tissue to prevent early degradation and shrinkage. Although the meniscus is a challenging but valuable structure, cell‐mediated treatment may support the preservation and/or rejuvenation of meniscus architecture and function, while augmenting long‐term outcomes of interventions for meniscus repair.^21^, ^22^ Accruing evidence suggests that human multipotent progenitor cells have therapeutic potential in orthopedics and that adipose‐derived mesenchymal stromal cells (aMSCs) are a viable option for cartilage regeneration, (fibro‐)chondrocyte differentiation, and meniscus engineering.^23^ Analogous to a cell injection technique developed for repopulating collagen meniscal implants (CMI),^24^ this proof‐of‐concept study examined the feasibility of injecting mesenchymal stromal/stem cells (MSCs) in meniscus allograft tissue, as well as established the injectable cell quantity and volume, survival, migration, and proliferation up to 28 days of incubation.

## 2 METHODS

### 2.1 Acquisition of donor material

**Figure 1:**
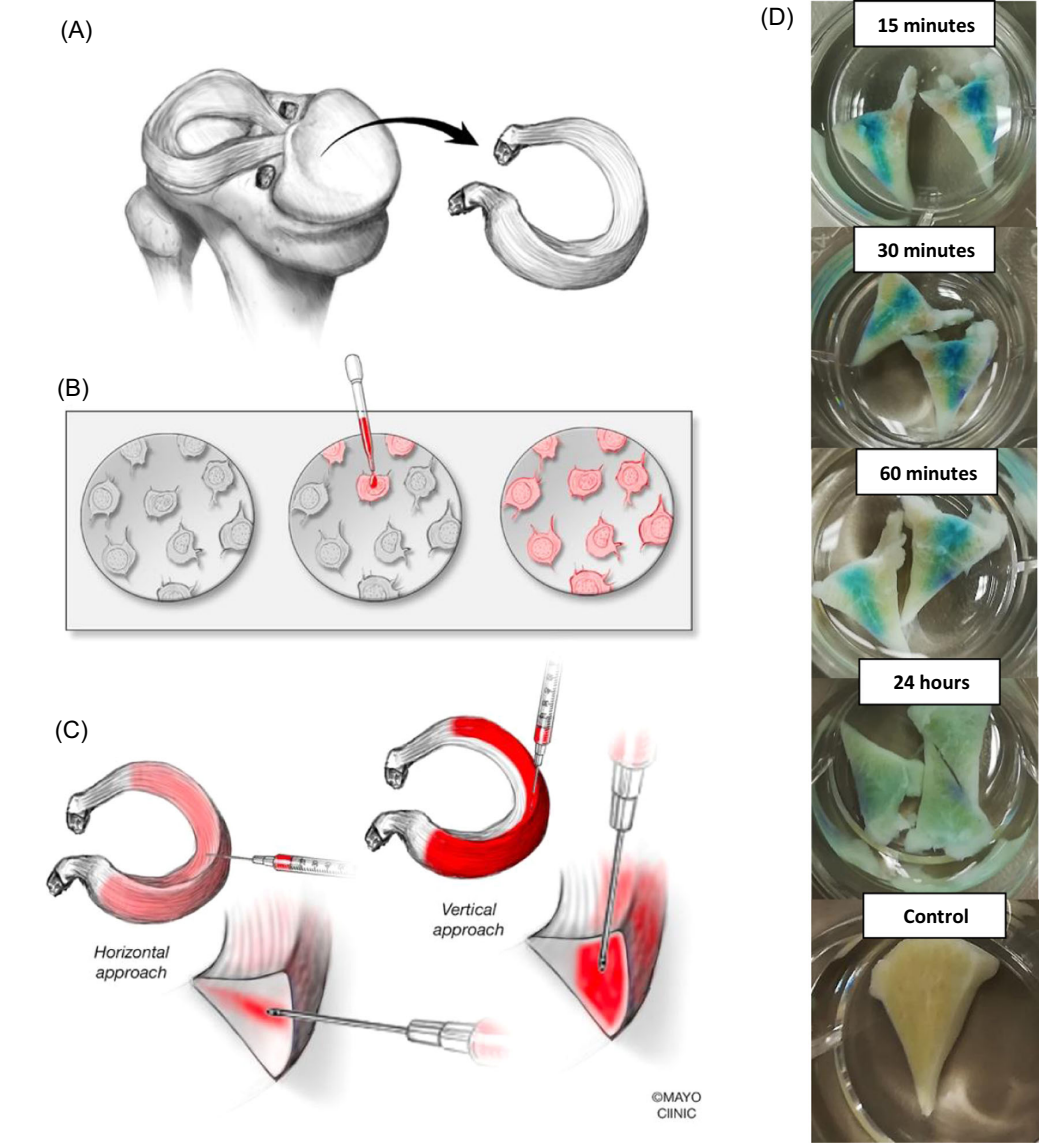
(A) Medial meniscus allograft. (B) Mesenchymal stromal cells labeled with Incucyte NucLight Rapid Red Reagent (1:500) (C) Horizontal and vertical injection approach. (D) Progressive stain expansion in the graft over 24 h after horizontal injections with toluidine blue (90:10 dilution) [Color figure can be viewed at wileyonlinelibrary.com]

Fresh‐frozen adult meniscus allografts (−80°C) attached to bone plugs (hemi‐plateau) were acquired through JRF ortho® (Centennial) (Figure 1A). Donor demographics and reasons for clinical transplant withdrawal were reported. Low‐passage human MSCs were provided by the Immune Progenitor and Cell Therapy lab (IMPACT) in Mayo Clinic. MSCs were obtained during liposuction from a single representative female adult donor. Written informed consent was obtained and collection procedures were performed based on IRB‐approved protocols. MSCs were harvested and expanded in a commercial platelet lysate product (PLTMax, Mill Creek Life Sciences) using the same protocol as described by Crespo‐Diaz and others.^25^ These MSCs have been extensively characterized by RNA‐seq and cell surface marker expression.^26^, ^27^ The mesenchymal cells we used to represent the immature perivascular stromal fibroblasts that have the potential to differentiate into multiple mesenchymal lineages in cell culture. Certainly, these cells are not related to embryonic pluripotent stem cells because they do not robustly express the pluripotency markers Sox2, Oct4/POU5F1, or Nanog.^26^

### 2.2 Cell and tissue culture

Cell and tissue cultures were performed in sterile hoods and certified incubators. For all experiments, advanced Minimum Essential medium (MEM; Gibco) was used and supplemented with platelet lysate (PLTMax, Mill Creek Life Sciences), l‐glutamine (GlutaMAX, Gibco), penicillin/streptomycin (Gibco), and 2 U/ml heparin. MSCs were expanded from one vial (±2.5E6 cells) and cultured until more than 80% confluency was reached. For all experiments, passage 4 cells were used. Cells were detached from monolayer with trypsin‐EDTA (Gibco) and counted in a hemocytometer chamber. Meanwhile, Ringer's lactate solution was preheated (37°C) to suspend the cells and bring them in a 1‐cc syringe for injection. Injected and control tissue samples were submerged in the above‐mentioned medium and incubated at 37°C with 5% CO_2_. A sterile medium change was performed every 3 days.

To distinguish injected MSCs from (dead) residing donor meniscus cells, labeling of MSCs was performed using NucLight Rapid Red Reagent (Essen BioScience) and cells were seeded in T75 flasks (seeding density 3000/cm^2^) with a final volume of 12 ml (1:500 dilution in supplemented advanced MEM medium) (Figure 1B). After 24 h, cells were detached with trypsin‐EDTA (0.05%, Gibco) and counted in a hemocytometer chamber.

### 2.3 Toluidine blue injections

To start, a single allograft was thawed and horizontally injected with toluidine blue stain (10% phosphate‐buffered saline [PBS]) using an 18‐gauge (G) needle and 1‐cc syringe to assess fluid dispersal of an injected solution in meniscus tissue. After injection, tissues were submerged in PBS and kept for 24 h in a 37°C rotating incubator. At 15, 30, and 60 min and 24 h postinjection, the injection site was macroscopically assessed on manual radial sections. This procedure informed our injection techniques, assessment of the average injected volume, and spreading of the stain within the tissue.

### 2.4 Injection with MSCs

The cell‐based injection experiments were subdivided in short‐term (Day 0 and Day 2) and long‐term (4, 14, and 28) assessments. Priorities in the short‐term experiments were (1) identification, localization, and spread of the injected cells, (2) assessment of cell viability and proliferation properties, and (3) quantification of MSCs immediately after injection. The main objective of the labeled cell experiments was the qualitative assessment of tissue slices for the distribution of injected cells.

#### 2.4.1 Short‐term experiments

First, three frozen medial meniscal grafts were brought to room temperature and allografts were radially cut into equal pieces of approximately 1 cm^2^. Labeled MSCs with Nuclight Rapid Red Reagent were resuspended in a medium loaded in a 1‐ml syringe. Plain cells, that is, cells that are unlabeled, were brought in suspension with preheated lactated ringers in a 1‐cc syringe. In total, 12 pieces were injected vertically (in a femorotibial direction) with approximately 100‐µl cell suspension using a 21G needle (Figure 1). Four pieces were injected with labeled cells with two pieces a dose of approximately 0.7E6 cells and two pieces 1.3E6 cells, respectively. The remaining pieces were injected with 0.6E6 plain MSCs. Control samples were injected with acellular PLTMax 5% media containing only the NucLight Rapid Reagent (1:500). Immediately (Day 0) after injection, samples with labeled MSCs were processed with cryosections (each 8th slice was mounted), whereas plain cell samples were embedded in paraffin, stained, and imaged, as described earlier. The second pair of plain MSC samples was used for fresh live/dead staining on Day 0 and after 2 days of incubation, with each accompanied by one of the acellular control samples.

Similarly, for direct cell quantification, four meniscus pieces were injected with plain expanded MSCs (passage 4) using a 20G needle, two vertically and two horizontally, with each injection containing approximately 100‐µl cell suspension. One vertically injected control sample and one horizontally injected control sample were provided with acellular lactated ringers. Samples were immediately fixed after injection and followed by the paraffin‐embedding protocol. Entire tissue samples were sectioned perpendicular to the direction of injection. Staining with hematoxylin and eosin (H&E), Safranine‐O, and Masson Trichrome, and immunohistochemistry (IHC) for markers Ki67 and alpha‐smooth muscle actin (α‐SMA) were performed as described earlier. The presence of cells was initially screened at ×5 magnification. If cells were present, images were taken at ×20 magnification to permit manual cell counting and evaluation with the ImageJ application. Total cell counts for each slide were calculated and averaged for two consecutive slides.

#### 2.4.2 Long‐term experiments

For long‐term experiments, the goal was to determine (1) MSC survival in the graft, (2) the cell migration pattern, and (3) the proliferation ability of injected MSCs. Two medial menisci were cut in half and the posterior part was used to inject. With an 18G needle, seven injections were placed in a horizontal way from the outer synovial area toward the inner (Figure 1C). During each needle punch, approximately 100‐μl cell suspension was injected. The second meniscal half was injected with preheated lactated ringers only (no cells) and served as a negative control. On Days 4, 14, and 28 of incubation, one or more injection sites were cut manually in radial sections for fresh live/dead stain with confocal imaging. Paraffin‐embedded histology was performed with H&E stain and IHC for the protein Ki‐67 on Day 14.

### 2.5 Histology

For histological analysis, samples were either paraffin‐embedded or visualized by cryosections. Tissues were first fixed with 10% buffered formalin for 5–7 days, after which stepwise dehydration with increasing ethanol concentrations was performed. Samples were cleared in xylene and immersed with paraffin for a minimum of 4 h in a 60°C oven. After mounting, tissues were entirely sectioned on a microtome at 10‐µm thickness and stained with Mayer's H&E Y (Sigma),^25^ Safranin‐O (Sigma‐Aldrich),^28^ and Trichrome Stain (Abcam). Indirect IHC with 3ʹ‐diaminobenzidine clearance and hematoxylin counterstain was performed for the proliferation marker Ki‐67 (gene symbol MKI67; Cell Signalling Technology®) and α‐SMA (gene symbol ACTA2; Abcam). Samples from mouse liver were used as a a positive control. Stained histology slides were photographed on a standard light microscope (Zeiss Axio, Carl Zeiss Microscopy) and processed in ZEN software (Carl Zeiss Microscopy). Cryosections were conducted on tissue samples with labeled MSCs using Cryomatrix embedding resin (Thermo Fischer Scientific) and a liquid nitrogen snap‐freezing method. Samples were cut in the axial plane (vertically injected) on a cryostat (Leica, CM3050S‐3‐1‐1; Leica Microsystems Inc.) at 25‐µm thickness. Imaging of these samples was performed using the LionHeart™ FX Automated microscope and Invitrogen™ EVOS™ FL Auto Imaging system. In addition, immunostaining with Hoechst 33342 (Thermo Fisher Scientific), Collagen‐1 (gene symbol COL1A1; Novus Biologicals), and Phalloidin‐iFluor reagent 647 (Abcam) was conducted and imaged with the Invitrogen™ EVOS™ FL Auto Imaging system in the respective 4′,6‐diamidino‐2‐phenylindole (DAPI), green fluorescent protein (GFP), and TxRed channels.

### 2.6 Live‐dead assay

LIVE/DEAD Kit for mammalian cells consisting of calcein AM and ethidium homodimer‐1 (Thermo Fisher Scientific) was used to determine MSC viability after injection. Working solutions were freshly prepared on the day of analysis and contained 10 ml PBS (Gibco) supplemented with 10 µl ethidium homodimer‐1 and 5 µl calcein AM. At the predetermined time after injection, tissue samples were manually cut in radial, longitudinal, or horizontal slices (depending on the injection direction) and subsequently submerged in working live/dead solution for 20 min in a dark tissue incubator (37°C with 5% CO_2_). Rinsing with PBS was performed twice for 5 min before imaging with confocal microscopy (ZEN Black software, ZEISS, channels 488 nm and CYP5).

### 2.7 Statistical analysis

For the cell labeling and quantification experiment, we worked with two samples per experimental group and took the average and standard deviation of the outcomes to describe statistical distributions. All analyses for differences and changes due to treatment are based on a subjective assessment of the data. The cell labeling results are provided as a qualitative illustration of the findings. The experiments were performed in duplicate to formally demonstrate that the results are replicable.

## 3 RESULTS

A total of seven adult meniscus allografts (5 medial and 2 lateral) were used for the experiments. None of the grafts exhibited any structural defects upon macroscopic screening.

### 3.1 Stain injection

The needle was horizontally introduced at the synovial attachment. While rotating gently, the needle was pushed toward the inner tip of the graft while exercising caution to avoid puncture of femoral or tibial contact surfaces. The most feasible approach for fluid injection was needle insertion followed by retraction coincident with fluid expulsion and rotation of the needle during retraction to avoid the generation of excessive pressure or fluid leaks. This technique permits the loading of 100 μl in the meniscus per injection site without incurring stain leakage. This volume was applied in subsequent cell injection experiments. After incubation, a blue horizontal trace was observed on radial sections, which gradually expanded over 24 h (Figure 1D).

### 3.2 Cell injections

#### 3.2.1 Short‐term experiments

**Figure 2:**
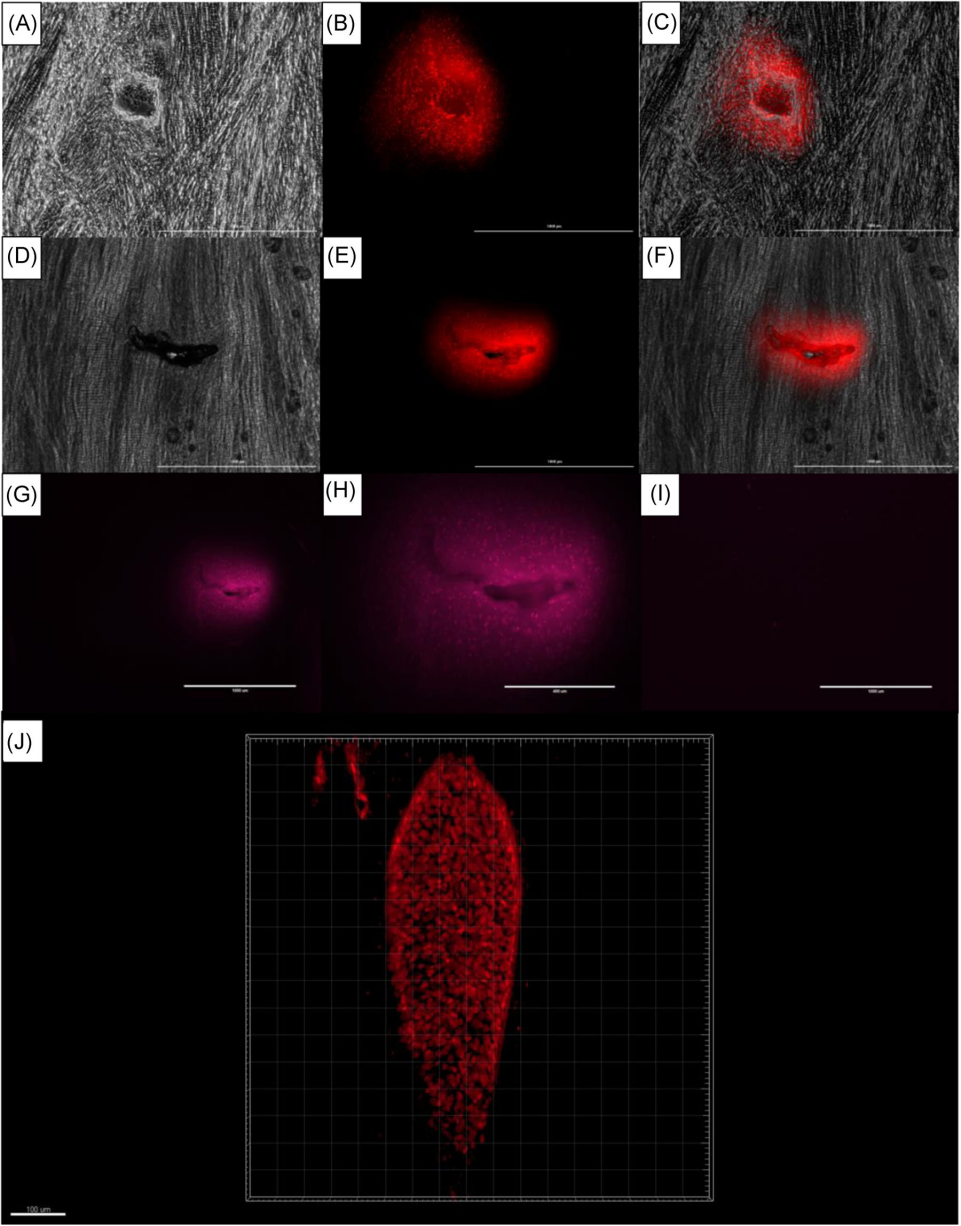
Allograft injected with labeled mesenchymal stromal cells (MSCs) (Incucyte® NucLight Rapid Red Reagent) and imaging with LionHeart™ FX and EVOS™ FL Automated microscopy and Confocal Microscopy (J): meniscus sample 1 (A)–(C) and meniscus sample 2 (D)–(I). (A) and (D) Phase contrast channel showing puncture site surrounded by well‐aligned collagen fibers of meniscus. (B), (E), (G), and (H) CY5 channel illustrating labeled and viable MSCs in a circular pattern around puncture site. (C) and (F) Phase contrast and CY5 channel superimposed to create this composite image. (I) control sample. (J) magnified image (CY5 channel on confocal microscopy) to illustrate that staining represents cell presence. Magnitude ×4: Scale bars 1000 µm (A–G, I). Magnitude ×10: Scale bars 400 µm (H). Magnitude ×10, scale bars 100 µm (J) [Color figure can be viewed at wileyonlinelibrary.com]

In the short‐term experiments (Day 0 and 2), doses for labeled cells were approximately 0.7E6 and 1.3E6 cells (100 µl) per injection site and approximately 0.6E6 of MSCs (100 µl) for unlabeled cells per injection site. Imaging of samples injected with labeled cells revealed a positive fluorescent signal on the CY5 channel, indicating the presence of MSCs in a circular pattern surrounding the puncture site (Figure 2). Mock‐injected samples without MSCs did not show a positive signal when imaged with the CY5 channel. Investigation of the distribution of labeled MSCs throughout the samples revealed that, on average, samples injected with the lower dose (with 0.7E6 cells) contained cells in 80% of the sample, whereas samples injected with 1.3E6 MSCs showed the presence of labeled cells in approximately 50% of sample. In addition, qualitative assessment of all images revealed that relatively more cells are present in samples injected with the lower dose.

**Figure 3:**
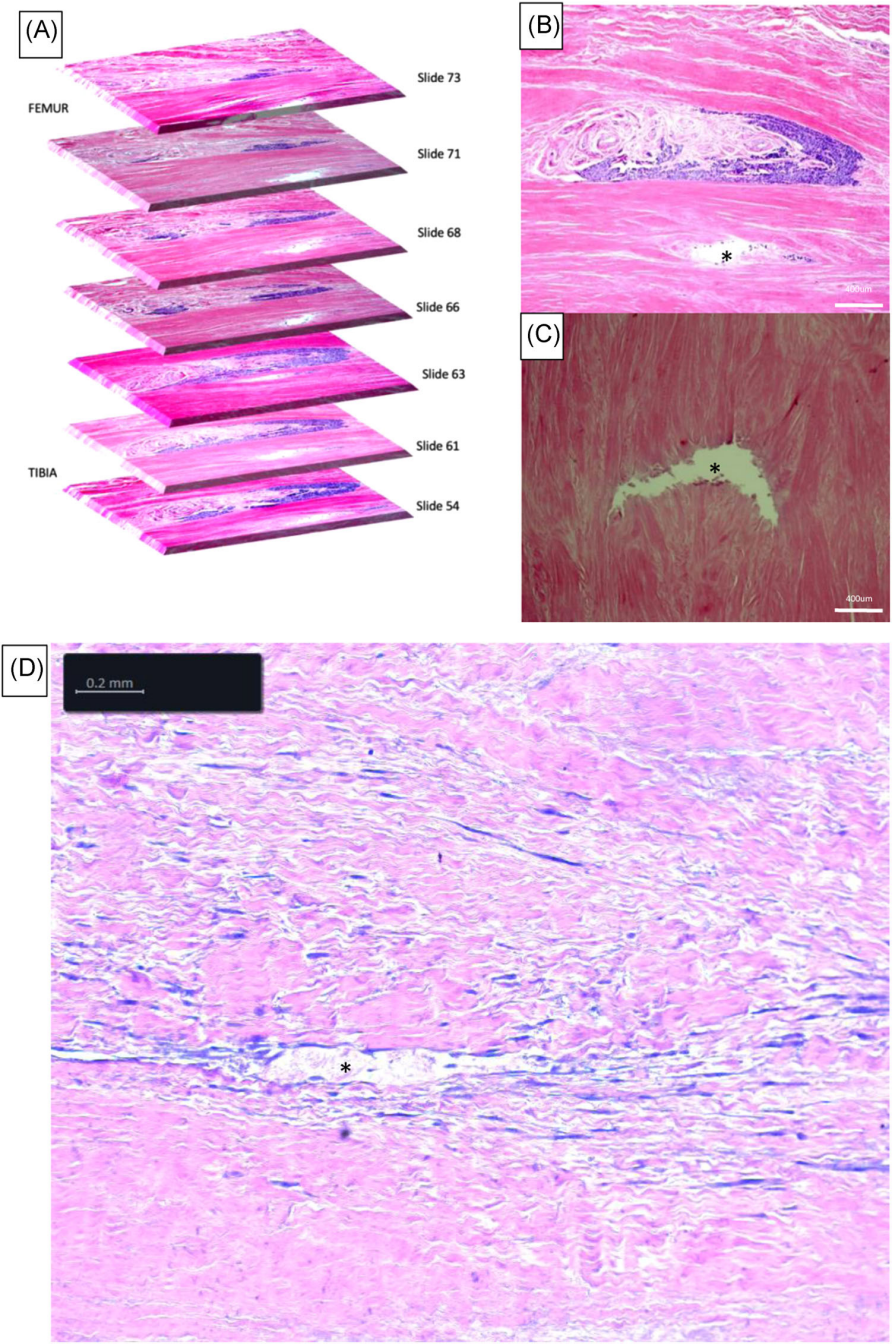
Meniscus allograft injected with mesenchymal stromal cells (MSCs) (A), (B)–(D) and control sample (C). Hematoxylin and eosin (H&E) stain. (A) Representation of full‐thickness slicing of sample. (B) Sample injected with MSCs showing a cluster of cells with larger cell nuclei, representing MSCs. (C) Control sample injected with acellular lactated ringers without the presence of MSCs. Injection site is marked with *. ×5 magnitude. Scale bars 0.4 mm. (D) Longitudinal meniscus section after 14 days of incubation showing the spindle‐shaped cell morphology and the migration pattern of MSCs in a densely packed collagen matrix. Scale bars 0.2 mm, ×10 magnification. Injection site marked with * [Color figure can be viewed at wileyonlinelibrary.com]

For the cell quantification experiments, samples were injected with approximately 0.6E6 of plain MSCs (100 µL) per injection site. Full‐thickness slicing of the four samples yielded an average of 486 tissue slices (SD 59) for vertical injections and 578 tissue slices (SD 81) for horizontal injections. With a slice thickness of 10 μm/section, these measurements corresponded with a height of 4.9 mm and width of 5.8 mm, resembling the macroscopic measurements of the graft samples (Table 1). H&E stain allowed for screening of the dense MSC clusters and counting was performed as described previously. In ImageJ, cell count resulted in 0.19E6 cells (±0.05E6) (32%) in the vertically injected samples and 0.40E6 cells (±0.02E6) (67%) in the horizontally injected samples (Figure 3). Control samples did not show typical cell clustering (Figure 3C).

**Table 1:** Cell count

		Total amount of tissue slices (= max. height/width of injection canal)	Tissue slices with MSCs	Ratio of tissue slices with aMSCs/total amount	Total amount of cells	Average standard deviation
**Vertical injection**						
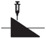	Sample 1	444 tissue slices	244 tissue slices	0.56	230,176	Mean: 192,764
	Sample 2	528 tissue slices	324 tissue slices	0.61	155,352	*SD*: 52 908
**Horizontal injection**						
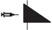	Sample 1	520 tissue slices	246 tissue slices	0.47	387,840	Mean: 401,130
	Sample 2	635 tissue slices	285 tissue slices	0.45	414,420	*SD*: 18 795

Abbreviation: aMSCs, adipose‐derived mesenchymal stromal cells.

**Figure 4:**
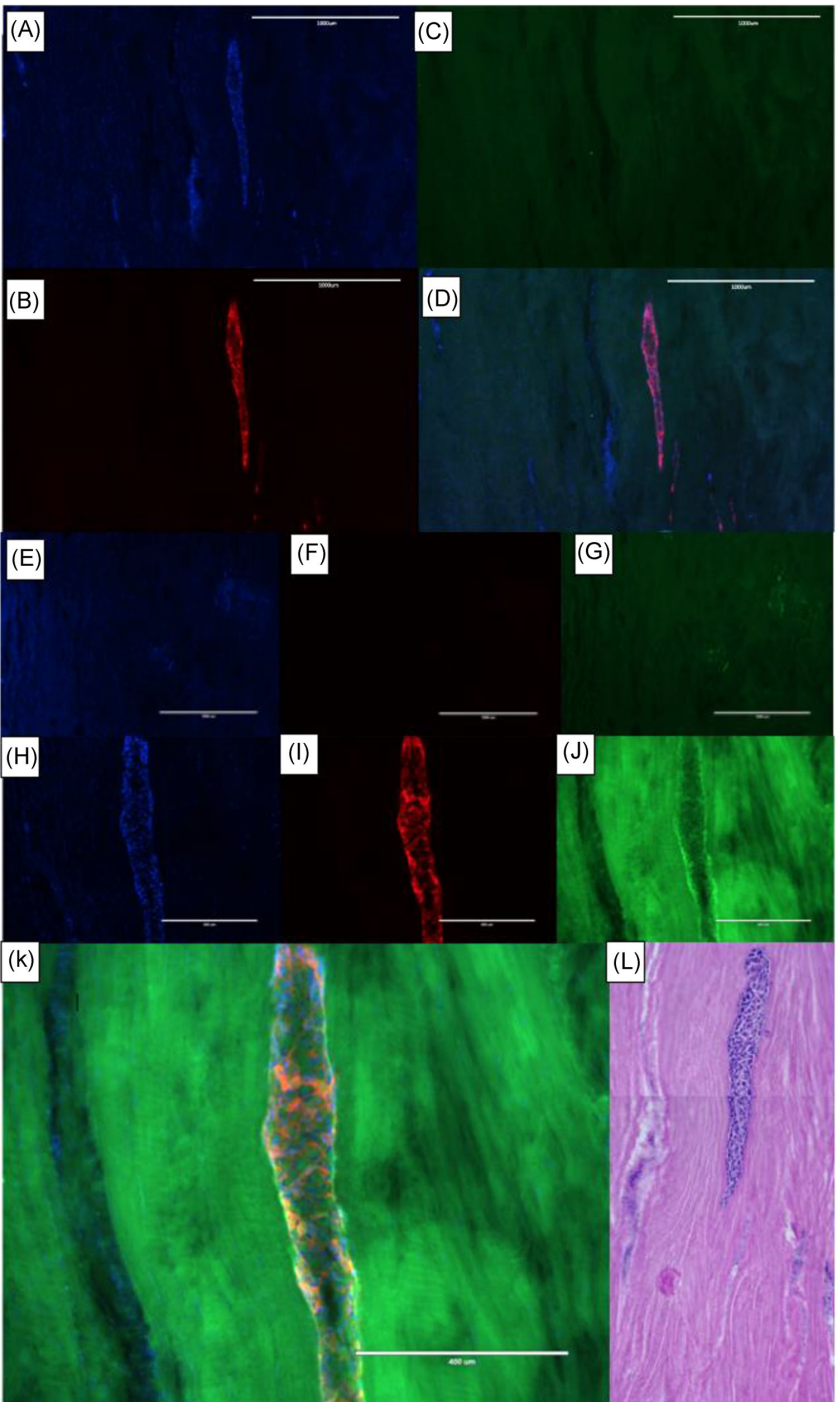
Meniscus allograft injected vertically with approximately 0.6E6 MSCs on Day 0. (A), (E), and (H) Hoechst 33342 nuclear stain (DAPI channel). (B), (F), and (I) phalloidin‐iFluor 647 (TxRed channel). (C), (G), and (J) Collagen type 1 (GFP channel) (D) and (K) channels superimposed to generate composite figures. (L) Hematoxylin and eosin (H&E) stain on consecutive tissue slice indicating that cluster of cells positive for phalloidin also has enlarged cell nuclei and a remarkably different architecture than surrounding meniscal tissue. (E)–(G) control sample: injected with lactated ringers (A)–(G) ×4 magnitude, scale bar 1000 μm. (H)–(K) ×10 magnitude, scale bar 400 μm. DAPI, 4′,6‐diamidino‐2‐phenylindole; GFP, green fluorescent protein; MSCs, mesenchymal stromal cells [Color figure can be viewed at wileyonlinelibrary.com]

**Figure 5:**
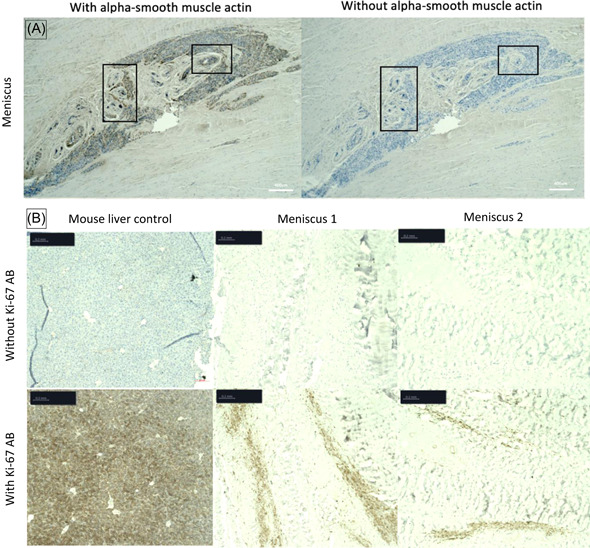
Standard immunohistochemistry (IHC) assay for alpha‐smooth muscle actin (alpha‐SMA) (A) and Ki‐67 (B) on paraffin‐embedded samples. (A) Positive staining for alpha‐SMA indicating that the circular structures (boxes) are vascular in nature. Mesenchymal stromal cells (MSCs) were mainly found around the vascular network in the meniscus, indicating perivascular homing of the cells. Tissues were sectioned in a horizontal way. Scale bar 0.4 mm. ×5 magnification. (B) Positive Ki‐67 marker to assess MSC proliferation 14 days after injection in a meniscal allograft. Mouse liver was used as a positive control sample to compare with two separately injected meniscus samples. IHC protocol without KI‐67 antibody (up) and with KI‐67 antibody (down). Tissues were sectioned in a vertical–longitudinal way. Scale bar 0.2 mm. ×10 magnification. AB, antibody [Color figure can be viewed at wileyonlinelibrary.com]

Immunostaining results showed cell nuclei (Hoechst 33342—DAPI channel) and organized alignment of collagen fibers (collagen‐1—GFP channel) throughout the whole tissue sample and control sample. Through Hoechst 33342 stain, several zones with high cell density in the synovial border were identified and one large spindle‐shaped cluster with a higher cell density in the middle of the sample, which was not present in the control sample. Staining with phalloidin for intact cytoskeletons displayed a positive fluorescence signal (TxRed channel) limited to these dense cell clusters. Phalloidin signal, which visualizes the intact actin cytoskeleton in live cells, was completely absent in control samples (Figure 4). The majority of H&E slides showed the presence of cell clusters with larger cell nuclei than neighboring cells derived from the donor meniscus in the sample center. These clusters were mainly found in the perivascular network and compressed longitudinally between collagen fibers as disc‐like structures (Figure 5A). Two consecutive tissue slices revealed that the cluster positive for phalloidin stain resembled the larger cell nuclei cluster with H&E (Figure 4K,L).

**Figure 6:**
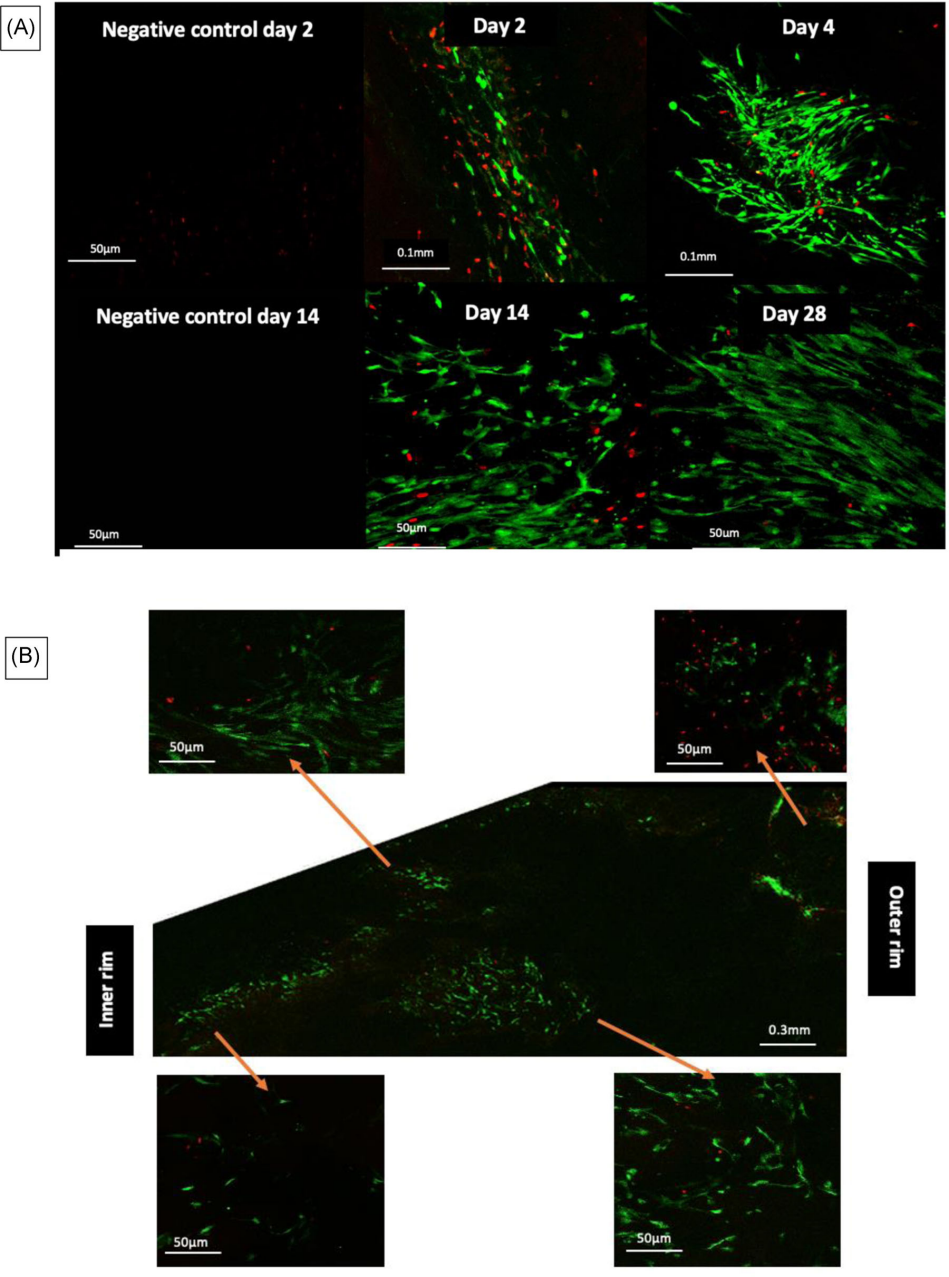
Live (green)–dead (red) stain images by confocal microscopy. (A) At the four predetermined timepoints after graft injection with mesenchymal stromal cells (MSCs) and their respective negative controls. On Day 14, no background dying native meniscus cells originating from the donor tissue were visible. (B) Radial meniscal allograft section 28 days after injection with MSCs. The tissue sample was incubated in an advanced Minimum Essential medium supplemented with 5% platelet lysate [Color figure can be viewed at wileyonlinelibrary.com]

Fresh live–dead staining immediately after injection showed a significant amount of viable (green) round cells at the injection site in a circular trend. After 2 days of incubation, a considerable number of living cells were observed but at a lower density. Dying (red) cells in microscopic images could either be injected MSCs or native donor meniscus cells, as indicated by the control sample (Figure 6A). Although the red fluorescent signals cannot discriminate between native and exogenous cells, we conservatively assume that at least some of the observed signals detected here represent injected cells that perished. Yet, surviving MSCs with a fibroblast‐related spindle‐like cell morphology were clearly detected on Day 2 (Figure 6A).

#### 3.2.2 Long‐term experiments

Viability of injected cells was confirmed at 4, 14, and 28 days of incubation with unlabeled MSCs (Figure 6A). From Day 2 onward, the majority of living cells adopted a typical fibroblastic spindle‐like morphology, as observed by live–dead staining in confocal microscopy, which was distinct from the original round/oval shape directly after injection (Day 0). On Day 14, the control sample injected with acellular lactated ringers showed no signal in the CYP5 channel, which reflects the absence of cell death (Figure 6A). Hence, there appears to be no active cytonecrosis of the original meniscus cells residing in the allograft. Rather, red signals in the test sample are attributable to the MSCs that were injected. On Day 28, the live/dead ratio was estimated as 80%–85% by the assessed images, whereas a spread of living cells was observed beyond the injection site (Figure 6B). Histological analysis on Day 14 revealed a disc‐like longitudinal migration pattern of the injected MSCs in alignment with the densely packed circumferential collagen fibers (Figure 3D). IHC for proliferation marker Ki‐67 was positive in the tested MSC samples at this time (Figure 5B).

## 4 DISCUSSION

The most important findings of this study are two‐fold. First, injecting a defined volume (100 μl) and amount of MSCs (>0.1 million) in a fresh‐frozen meniscus allograft is feasible and potentially translatable in a clinical setting, because cells retain viability and are present throughout the injection site immediately after injection. Second, injected fresh‐frozen allografts can be cultured for 14–28 days postinjection, with cell survival rates more than 80%. The administered cells maintain the ability to proliferate and migrate according to the orientation of the circumferential collagen bundles.

The findings of this study indicate that it is feasible to inject cells into a meniscus allograft, resulting in the presence of viable cells capable of migration and proliferation, regardless of spatial constraints imposed by the dense extracellular matrix of the meniscus. Nuclear and cytoskeletal labeling of viable cells, combined with histological staining, permitted identification of MSCs throughout the injection site of the graft at the earliest time point (i.e., Day 0). Confidence in our findings is based on the detection of distinct cell clusters with larger cell volumes and nuclei, which represent typical morphological features of MSCs that were present only in injected grafts and clearly different from surrounding tissues.

Furthermore, live–dead stain assays on Day 0 and Day 2 after injection and IHC of Ki‐67 (as proliferation marker) on Day 0 and especially on Day 14 confirmed cell viability and indicated that the MSCs were actively dividing in the samples and did not enter cellular quiescence after injection into the dense microenvironment of the allograft (Figure 5B).

Interestingly, freshly injected cells were mainly found around the vascular network in the meniscus (Figure 5A). The perivascular area mainly consists of adipose and loose fibrous tissue, a low‐resistance site where the MSCs logically may end up when injected under pressure between dense collagen fibers. Alternatively, the extracellular matrix of the meniscus and the stroma around blood vessels in decellularized allografts may still contain latent growth factors (e.g., attached to glycosaminoglycans and ECM proteins) that are released upon remodeling of the tissue in response to the injection of MSCs. It is conceivable that a gradient of previously deposited factors may exist that radiates from the original blood vessels. This putative gradient could potentially provide directionality (e.g., through chemotaxis) and support preferential homing of cells to the perivascular niche around blood vessels.

Long‐term live–dead assessments showed abundant MSCs (green) in the area of injection site on Days 14 and 28 (Figure 6). By confocal microscopy, the MSCs had a spindle‐like appearance embedded in the dense meniscus matrix as early as Day 2 in contrast to their round/oval shape directly after injection. However, some dying cells (red) were observed as well, which might have been part of an outbalanced biological process between new cell divisions and cell death. Mechanisms responsible for the apparent change in MSCs phenotype (round to spindle‐shaped morphology) may be influenced by many different physical and chemical stimuli such as the introduction to the dense microenvironment of the meniscus. In addition, while the tissue is decellularized (“dead”), the ECM is expected to have residual bioactive proteins (e.g., bound to glycosaminoglycans associated with ECM proteins) that may instruct how MSCs behave upon injection. Moreover, based on histological analysis at 14 days after injection, MSCs appear to expand in a horizontally aligned fashion as they were initially injected (Figure 3D). Both on H&E and Safranin‐O stain, cells were oriented in a similar way as the circumferential meniscus collagen fibers were aligned. Interestingly, cells were observed distal to the initial injection channel by horizontal dispersion; few or none appeared to migrate across the vertical plane.

To optimize injection procedures relative to the lamellar organization of the meniscus, we examined both horizontally and vertically injected allografts for the presence of injected cells. We observed that the distribution of cells throughout the injection site differed modestly between vertically injected cells (i.e., cells detected in ~60% of tissue slices) and horizontally injected (cells present in ~46% of samples. Subsequent experiments with pre‐labeled cells that were injected vertically in the graft revealed improvement in the fraction of positive samples (~80%). Taken together, these experiments reveal that, on average, about 60% (range 46%–80%) of the samples exhibit positive cell staining, and that vertical injection may perhaps have slightly better outcomes than horizontal injections. We propose that as MSCs are able to migrate horizontally along the intrinsic lamella of the meniscus, it may be more beneficial to inject cells vertically. The vertical approach permits the initial deposition of cells in each lamellar compartment throughout the entire height of the graft initially, whereas the horizontal approach may limit injection to one or more meniscal lamella that may impede the vertical migration of cells. This proposed model would predict that vertical injection is more likely to result in a homogenous distribution of injected cells over time. Moreover, during meniscal allograft transplantation surgery, an additional set of sutures is placed through the graft, which is less likely to interfere with vertical injection channels as compared with horizontal ones. Regarding the technique of cell delivery, we note that in our experience, it was beneficial to rotate the needle while pulling back from the injection site. It is possible that rotation may mitigate backpressure and spillage, and this empirical knowledge was incorporated into our standard injection protocol.

Labeled cell experiments were done as part of initial dosing experiments to optimize delivery. The range of target doses we selected was based on the excellent work of the Hollander group on the repair of torn avascular meniscal cartilage using a seeding density of 1 million MSCs per square cm.^29^ Remarkably, injection of two different doses of labeled cells in samples revealed that higher cell dosing does not necessarily result in an increased presence of cells in the samples. Another noteworthy feature between the two groups of samples is that menisci injected with the lower dose of cells reveal a more distributed pattern of cells throughout the sample in comparison with the samples injected with the higher dose of cells, that is, the location of cells in high‐dose samples is limited to the injection site and present in 50% of tissue slices versus 80% of tissue slices in specimens injected with the lower dose. It is plausible that the higher cell dose increased the viscosity of the cell suspension in the injection device. In addition, samples injected with a higher cell dose might have less nutrients available, resulting in a decreased cell number. Also, cells were selectively absent at the top and bottom of each allograft perhaps due to leakage. Previous histological studies of allograft biopsies have shown that allograft repopulation is restricted to the superficial zone and that the center portion of the transplant biopsy did not exhibit the presence of cells.^30^, ^31^ Our injection strategy complements this previous study by showing that repopulation of the inner portion of the graft therapy is feasible and perhaps clinically relevant, because the inner tissue is normally less accessible but important for optimal restoration of the graft tissue. These observations in this study will guide future studies to define an optimal target dose, and our current assessment is that a dose of 0.6 million MSCs per injection represents a useful starting point for future studies.

Applications of stem cells for the purpose of meniscus regeneration have been described in other experimental models in which mesenchymal stem cells (MSC) were seeded on meniscal tears or scaffolds/grafts.^26^, ^27^, ^28^ Izuta and colleagues^32^ showed that MSC transplantation in the avascular portion of a meniscus leads to survival and proliferation of the cells as well as production of extracellular matrix (ECM), contributing to regeneration. Furthermore, several studies applied a seeding strategy of MSCs on meniscus scaffolds, leading to repopulation and detection of mRNA expression for aggrecan/ACAN and type X collagen/COL10A1.^28^, ^29^

Yet, to date, only one published in vitro study attempted to improve cellular repopulation of meniscus allografts. This study reported outcomes of MSC seeding on the surface of a decellularized needle‐punched meniscus allografts (28G and 1 mm spacing).^27^ After 28 days of tissue culture, seeded cells were infiltrating through the created channels, whereas nonpunctured seeded grafts appeared to remain empty. These results emphasize the formidable barrier that high‐density collagen fibers may pose for cell migration within meniscus grafts and the importance of increased graft porosity for spontaneous host cell influx. Moreover, the creation of space channels in the graft is somewhat similar to the meniscus “trephination” technique that has been described as an approach to induce blood supply for tear healing in the avascular meniscal zone.^30^

A review by Michiewicz et al.^33^ on preservation and sterilization methods of meniscus allografts discussed that fresh‐frozen meniscus allografts are the most frequently used type of allograft, even though there are potential concerns with this tissue. The rationale of this study is to restore fresh‐frozen meniscus allografts with living cells to rejuvenate the graft before implantation into the patient. However, the use of freshly stored allografts is increasing. Although such tissues already contain live resident cells, injection of stromal cells in fresh, viable meniscus allograft tissue may still be considered. Injection of MSCs in fresh grafts could potentially re‐enforce the viability of the fresh tissue, whereas injection into frozen meniscus tissue would restore the viability of the decellularized graft. However, MSCs injected into fresh meniscus allografts may interact with the native meniscus cells and both cell types may mutually alter their biological behaviors and/or cell survival.

The current study identified several technical and cellular limitations that are not insurmountable but will require resolution in the future. First, this study represents an in vitro study. As such, it does not provide information about how cell injection would work in the complex postsurgical in vivo environment after injection during which the injected grafts will be subjected to tissue healing responses in the context of inflammatory and biomechanical processes. Second, this study primarily describes the feasibility of injecting MSCs in a meniscus allograft as a proof of concept. Upon completion of the first set of experiments that measured multiple in vitro outcome parameters, we repeated key parts of the study to ensure this study can be replicated in the hands of different investigators. However, duplicate outcome measures (*n* = 2) do not permit statistical analysis and all outcome assessments were based on subjective assessment of the data. Hence, rather than definitive quantitative proof for a new methodology, our study merely illustrates an exciting proof of concept that vertical cell injections of meniscus allografts are qualitatively possible while inspiring future studies on MSC‐mediated augmentation of meniscus repair.

Cell suspension leakage during injection was difficult to assess in each experiment. The actual cell count demonstrated that approximately two‐thirds of cells were not delivered in tissues injected vertically, whereas this could be minimized to one‐third for horizontal injections. Nevertheless, an important number of cells/injection site (> 0.1E6) was still loaded in the meniscus applying either both direction. As graft samples were not decellularized, live–dead stains < 14 days postincubation showed both dying residing meniscus cells and dying injected MSCs in red. On the basis of the red cell density and location, a gross distinction could be made between both, but exact cell survival immediately after injection could not be rendered. On live‐dead control samples more than 14 days, dying meniscus cells appeared to be degraded and were not visible anymore (red), which facilitated survival assessment of the injected cells only.

To minimize matrix damage that could compromise the mechano‐physiological properties of the grafts, we investigated the feasibility of injecting cells into the meniscus by testing different needle sizes between 18 and 27G and opted for the smallest needle size (21G) that still permits efficient injection of MSCs. Therefore, this size (21G) was used for all subsequent experiments. The effect of needle passage under moderate pressure on cell proliferation and survival was not assessed. However, Onishi et al.^34^ showed that the MSC viability, proliferation, and metabolism function are not affected by needle passage as small as 30G. As fluid pressure is exponentially related to the radius of the needle, we are confident that needle sizes 18, 20, and 21G selected for the majority of the experiments were not modifying the cells by mechanical sheering of fluid pressure.^34^ In addition, MSCs injected into meniscus tissue encounter backpressure from injecting fluid into a dense meniscus, and it is still uncertain whether this could have altered the effective dosing of MSCs upon injection. We have tested fluid pressure upon injection with a manometer but observed that the resulting data were highly variable, thus precluding the definition of a maximal limit on the observed fluid pressure during meniscus injection.

Finally, samples were not biomechanically tested after injection. Mechanical characteristics and material properties of the graft could potentially be compromised depending on the bore size of the needle and the number of injections. This feasibility study was solely targeting the biological cell performance in the graft; however, biomechanical analysis of cell enhanced meniscus grafts will be essential for any path towards clinical application.

The current study results are opening careful perspectives for clinically augmenting the initial viability of meniscus allografts, aiming to minimize the risk of graft shrinkage, optimize biological integration, and prolong MAT durability. Strikingly, MSCs were able to migrate following the alignment of collagen bundles in the meniscus, which provided a very encouraging avenue for consideration of MSCs in cellular repopulation of meniscus allografts in facilitating knee repair. Whether injected MSCs promote tissue recovery and synthesize relevant matrix proteins is a critical next step of this study. Although this study yielded encouraging study results, many factors remain to be clarified before this technique can be translated to in vivo models.

## 5 CONCLUSION

The study demonstrated that injection of more than 0.1 million MSCs is clinically feasible and may suffice in improving the biological properties of meniscus allografts. Cells maintain proliferation and migration properties for at least 14 days postinjection and survival rates of (>80%) after 28 days are observed. These findings attest to the feasibility of repopulating meniscus allograft tissues with viable cells that may accommodate biological integration after joint surgery.

## CONFLICT OF INTERESTS

Allan B. Dietz and Mayo Clinic have contractual rights to receive royalties from the licensing of PL technology to Mill Creek LIfe Sciences. In addition, Mayo Clinic and Allan B. Dietz hold equity in the company to which the technology is licensed. This conflict is managed according to policies and procedures regarding conflict of interest at Mayo Clinic. Other authors declare to have no conflict of interests.

## AUTHOR CONTRIBUTIONS

Caroline Struijk, Wouter Van Genechten, Peter Verdonk, Aaron J. Krych, Allan B. Dietz, Andre J. van Wijnen, and Daniel B. F. Saris collectively contributed to the study conception and design. Experiments and data collection were performed by Caroline Struijk and Wouter Van Genechten. The manuscript was written by Caroline Struijk and Wouter Van Genechten in consultation with Andre J. van Wijnen and Daniel B. F. Saris. All authors provided edits and comments on the manuscript and approved the final submitted manuscript
